# Analysis and optimization of the landing zone parameters of a sclera lens model

**DOI:** 10.5935/0004-2749.2023-0332

**Published:** 2024-12-26

**Authors:** Luiz Formentin, Yandely Chihuantito Choquechambi, Natalia Pereira Felix de Araujo, Samantha de Albuquerque Mori Miyazawa, Helena Maria Costa Oliveira, Rodrigo Teixeira Santos

**Affiliations:** 1 Ophthalmology and Visual Sciences Department, Escola Paulista de Medicina, Universidade Federal de São Paulo, São Paulo, SP, Brazil

**Keywords:** Contact lenses, Lifting, Keratoconus, Rehabilitation

## Abstract

**Purpose:**

This study aimed to modify scleral contact lenses to achieve a desired compression standard and to evaluate the effectiveness and reliability of the adjustments.

**Methods:**

In this nonrandomized, noncomparative, and partially masked study Scleral contact lens fittings were analyzed in 20 eyes of 12 patients (50% women, 50% men) diagnosed with keratoconus. Participants were selected based on their need for scleral contact lenses (SCLs), which was determined in complete ophthalmological examinations. Patients were tested with Zenlens scleral contact lenses (Bausch & Lomb, Vaughan, Ontario, Canada). We evaluated compression in the lens support area after one hour of use, excluding cases of peripheral lifting. Photos of the adaptations were sent to five experts for analysis of the quadrants (nasal, temporal, superior, and inferior). We used Fisher’s exact test for statistical analysis.

**Results:**

The proposed adjustment was highly effective (93.5% correct) in lens delivery (BL=0), with the interrater agreement between doctors ranging from 68.8% to 80.9%.

**Conclusion:**

The clinical parameters proposed for scleral contact lenses adjustment proved useful and reproducible, enabling their practical application to scleral lens adaptation.

## INTRODUCTION

There have been numerous technological advances in scleral contact lenses (SCLs), including improved covering materials, designs, and manufacturing techniques. SCLs have seen a marked resurgence after nearly a century of relative dormancy^(1^,^2)^.

In 1983, Ezekiel published the first description of lenses made from materials with greater gas permeability (rigid gas permeable [RGP]), which significantly reduced the risk of hypoxic complications of daily SCL use^(3)^ seen with polymethylmethacrylate (PMMA) lenses. This caused renewed interest in the potential use of SCLs^(4^-^6)^.

Further improvements built on this lens model, and the evolution of computerized lathes allowed highly complex designs. Among the most significant advances to which this has led are the development of prolate and oblate lens profiles, and the ability to make lens adjustments to sagittal height (SAG), limbal curve height, and the support area. Studies have identified a high prevalence of scleral asymmetry, and this can have a significant impact on proper lens fit. Precise alignment of the scleral lens support with the ocular surface is extremely important for properly fitting lenses^(7)^.

Furthermore, there are several variations of the corneoscleral transition. Barnett et al. have found significant differences in the corneoscleral junction (CSJ), which is greater along the horizontal than the vertical meridian. CSJ angles vary between the four quadrants, being the most pronounced in the nasal, followed by the inferior, superior, and temporal quadrants. The flatter scleral curvature seen in the latter may explain the clinically observed temporal decentering of lenses^(8)^.

Adjustments to the support area are fundamental to patient comfort and this increases lens wear time, as it is estimated that approximately 10% of SCL wearers discontinue their use due to discomfort^(9)^. This discomfort can be attributed to several causes, including poor scleral landing zone fit. Wearing spherical SCLs can cause patients to squint, decentering the lens to a specific area, usually the quadrant in which the lens does not align properly with the sclera^(10)^.

To deepen our understanding of the optimum adjustment requirements for SCLs, the main objective of this study was to evaluate the landing zone areas of SCL wearers for compression and identify possible SCL corrections. In so doing, we hoped to identify adjustment patterns that could be easily found using slit-lamp analysis.

## METHODS

This was a nonrandomized, noncomparative, and partially masked study. SCL fittings were evaluated in 20 eyes of 12 patients, including 6 women (50%) and 6 men (50%). All of the patients had keratoconus. After undergoing a complete ophthalmological examination, each participant was advised to wear contact lenses. Each patient underwent a scleral lens adaptation test due to a clear indication for SCLs, an ocular surface disease, or the failure of rigid gas permeable (RGP) corneal contact lenses to meet their optical needs.

This study was conducted in accordance with the tenets of the 2013 revision of the Declaration of Helsinki and approved by the Ethics Commission for Analysis of Research Projects CEP/UNIFESP (1340/2017; CAAE: 79411117.8.0000.5505). All patients were fully informed of the study aims and procedures and gave informed consent to participation.

The tests were performed using Zenlens scleral lenses (Bausch & Lomb, Vaughan, Ontario, Canada). These are SCLs with a multicurve design that allows personalized but standardized adjustments to the anterior and posterior surfaces of the lens. Possible modifications include adjustments to the anterior toricity, the limbal release curve (LCC), the flexion control, the microvault, and the advanced peripheral system^(11)^.

Patients were initially left wearing new SCLs for an hour and then each eye quadrant was evaluated to determine the levels of compression at 0, 1, 2, 3, and 4. Previous research has shown that the ideal waiting time before the initial evaluation of SCLs is 30 to 40 minutes. We chose to wait 60 minutes to ensure the fullest adaptation^(12)^. There were some cases in which, instead of compression, there was lifting of the periphery; however, as these were exceptions, we did not include these cases in this study. For each level of compression, a specific adjustment was made, as shown in Table 1^(13)^.

**Table 1:** Classification of the compression levels requiring SCL support, and our proposed corrections

Compression level	Parameters	Correction
**0** Ideal standard	No compression at all	No adjustment needed
**1** Mild compression	Difference in color without vessel interruption	FLAT 2 (60 microns)
**2** Moderate compression	Difference in color and interruption of some small vessels	FLAT 4 (120 microns)
**3** Intense compression	Compression with interruption of larger vessels	FLAT 6 (180 microns)
**4** Very intense compression	Compression with interruption of larger vessels, more acute, more at the edge	FLAT 10 (300 microns)

SCLs, scleral contact lenses.

**Figure 1:**
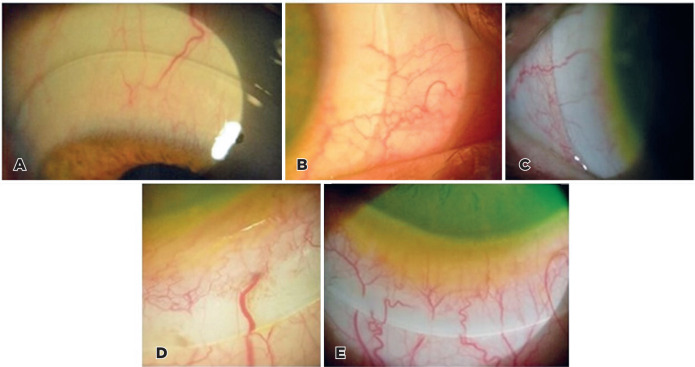
Photographs of compression levels of different severity in eyes requiring scleral contact lenses. A) Illustrates the ideal scleral support pattern, with no compression. B) Shows mild compression, characterized by a difference in color without interruption of the vessels. C) The compression is moderate. There is a difference in color and interruption of some small vessels. D) Shows intense compression with the interruption of larger vessels. E) The compression is very intense, more acute, and with interruption of larger vessels. The compression is also closer to the edge.

We then sent photos to five experts of each quadrant (nasal, temporal, superior, and inferior) of each eye before and after the adaptations. Photos corresponding to the compression classifications were collected in a spreadsheet. Figure 1 shows photos of eyes classified as A to E according to compression level. It is important to highlight that the experts were blinded as to whether each photo was taken before or after lens adaptation, which photos corresponded to which patient, or whether each photo was of a left or right eye.

### Statistical methodology

Comparisons of the distribution of correct classifications (a classification equal to zero at lens delivery [BL]) were carried out using Fisher’s exact tests (more than 20% of cells in a contingency table with expected values lower than five).

For all statistical tests, p-values <0.05 were considered significant. Analyses were performed using SPSS Statistics for Windows, version 20.0 (IBM Corp, Armonk, NY, USA) and STATA, version 17 (StataCorp. LLC, College Station, TX, USA) software.

## RESULTS

Our statistical analyses addressed two main points: the effectiveness of the adjustment proposal and the level of agreement between doctors using the proposal.

As can be seen in Table 2, 93.5% of the 77 postadjustment photographs (three of 80 photos were discarded due to poor quality), had a BL equal to zero, indicating that the correct adjustment had been made. This result was not heterogeneous according to the classifications initially assigned (p=0.072). Additionally, it was found that, among the cases in which no adjustment was required or made, only one (20%) showed no reduction in compression severity (and corresponding classification) at the initial assessment (test=1 and BL=1). Attached (attachments 1, 2, 3, and 4) are some examples of the support relationship observed in the test of eyes with adjusted lenses. With both the trial lenses and the customized lenses, the following parameters were used: diameter, 16 and 17 mm; deep sagittal, 4,500 up 6.100 microns; back curve, 6.4 up 9.0 mm; prolate and oblate design; landing zone up to +300 microns in each quadrant.

**Table 2:** Results of using the scleral contact lens classifications

Classification -test	Lack of fit n (%)	Hit n (%)	p-value
Total	5/77 (6.5)	72/77 (93.5)	0.072
Peripheral survey	0/3 (0)	3/3 (100.0)	
No compression	0/30 (0)	30/30 (100.0)	
Light compression	1/15 (6.7)	14/15 (93.3)	
Moderate compression	2/19 (10.5)	17/19 (89.5)	
Intense compression	1/8 (12.5)	7/8 (87.5)	
Very intense compression	1/2 (50.0)	1/2 (50.0)	

p-value -descriptive level of Fisher’s exact test.

Table 3 presents the percentages of correct answers per assessing doctor in relation to the classifications of the referring doctor. Only cases with a BL equal to zero were considered, excluding the “peripheral survey” category. In the expert evaluations, classifications that agreed with our own or differed by just 1 level (above or below) were considered acceptable. Using these criteria, the percentages of correct answers ranged from 68.8%-80.9%, indicating good accuracy.

**Table 3:** Numbers and percentages of correct contact lens classifications per independent assessor

	Doctor 1	Doctor 2	Doctor 3	Doctor 4	Doctor 5
Hit (up to 1 level of deviation)	44/64 (68.8%)	54/69 (78.3%)	54/69 (78.3%)	47/68 (69.1%)	55/68 (80.9%)
Exact hit	24/64 (37.5%)	28/69 (40.6%)	30/69 (43.5%)	28/68 (41.2%)	29/68 (42.6%)

Expert classifications no more than one level above or below the correct classification were considered to be of acceptable accuracy.

**Figure 2:**
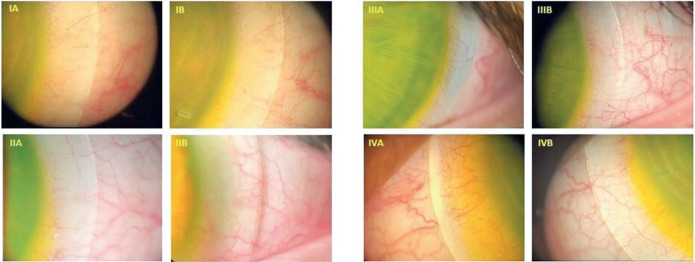
Photographic comparisons of the clinical effects of test scleral contact lenses and delivered scleral contact lenses. The A images correspond to the test lenses and the B images to the delivered lenses (BL). Image pairs IA and IB, IIA and IIB, and IIIA and IIIB show the temporal quadrant of the left eye. Images IVA and IVB show the temporal quadrant of the right eye. The trial lens in image IA caused slight compression, characterized by a difference in color. However, there is no difference in color or interruption of vessels in image IB of the same eye with the delivered lens. An ideal pattern is maintained. Image IIA displays moderate compression in the eye with the test lens, with disruption of small vessels. In delivered lens image IIB, an ideal pattern is maintained. In test lens image IIIA, there is intense compression, with interruption of larger vessels. However, in delivered lens image IIIB, an ideal pattern is apparent. In the test lens image IVA, the compression is very intense with marked interruption of larger vessels, especially at the edge, resulting in a more acute and pronounced color difference. In contrast, the IVB image of the eye with the delivered lens displays an ideal pattern.

Figure 2 compares the preand postadjustment clinical outcomes in the area covered by the SCL landing zone.

## DISCUSSION

The use of SCLs can change a patient’s life, reestablishing the vision necessary for their work and leisure activities, either by improving visual acuity or by protecting the cornea in ocular surface diseases. Naturally, the longer the duration for which a patient can wear their lenses, the better, and this depends on lens safety and comfort^(8^,^9)^.

Barnett et al. have demonstrated that quadrant-specific lenses provide improved fit, reducing the number of patients reporting a need to remove their lenses during the day. This discovery shows the importance of using lenses with specific support areas for better patient-specific adaptation and comfort^(8)^. Ritzmann et al. found that scleral asymmetry begins at the limbus, which is the most symmetrical area, and increases toward the extraocular muscles. Asymmetrical variation can influence the fit and stability of SCLs, as each lens must be adapted to the individual scleral curvature. Understanding these scleral characteristics and their variations is essential for adequate adaptation that ensures the comfort and effectiveness of each lens^(14)^. To increase success with SCLs, adjustments to the support zone should be as accurate as possible. Although there are now devices able to approximate the scleral anatomy of the cornea^(15)^, they are costly and the perfect fit still depends on slit-lamp analysis. To our knowledge, this is the first study to relate clinical presentation to the degree of adjustment required per quadrant.

To perform this study, we observed the reactions of blood vessels to the SCL landing zone. We observed five stages: 1) no pressure; 2) light pressure with a difference in color between the free conjunctiva and sub-SCL; 3) moderate pressure, as for stage 2 plus obstruction of small vessels; 4) intense pressure, as for stage 2 plus obstruction of medium vessels; 5) very intense pressure, obstruction of medium vessels and an acute pattern.

Based on our compression classifications, we adjusted the support zone to establish the optimal resistance of the part of the SLC facing the sclera based on the importance of good lens support in the conjunctiva/sclera. By adjusting the symmetry and landing zone of the SCL we established the corrections most suited to each classification. The SCL customizations were successful in around 90% of cases. We then considered whether the system was viable for use by other ophthalmologists. At this point, we mashed up photos of the landing zone (support area) before sending them to five ophthalmologists for evaluation. For each lens, we obtained four photos, one for each quadrant. There were two lenses used with each eye, a trial lens and a custom lens, amounting to eight photos per eye. As we treated 20 eyes, we obtained 160 photos. After these were merged, they were sent to five ophthalmologists for compression level classification to determine the level of agreement with our own classifications and the practical validity of these classifications and customizations. The interrater agreement levels were good, indicating that our system offers a feasible approach to improved SCL fitting.

A limitation of this study was the small number of cases with intense compression. Also, a possible problem with the proposed approach is the difficulty of reproducing images from slit lamps. Agreement levels may be higher when the system is used in real-world situations.

All of the participants in this study had keratoconus and received the same SCL model as we were concerned only with the landing zone. Nevertheless, our findings may be applicable to other scleral lens models and patients with different corneal diseases such as pellucid marginal degeneration or postcorneal transplant adaptation, but further studies are needed to investigate this. The clinical parameters proposed for scleral support area adjustments proved useful and reproducible, supporting their future use in scleral lens fitting practices.

## References

[1] Rosenthal P. (1986). The Boston Lens and the management of keratoconus. Int Ophthalmol Clin.

[2] Romero-Rangel T, Stavrou P, Cotter J, Rosenthal P, Baltatzis S, Foster CS. (2000). Gas-permeable scleral contact lens therapy in ocular surface
disease. Am J Ophthalmol.

[3] Ezekiel D. (1983). Gas permeable haptic lenses. J Br Contact Lens Assoc.

[4] Ruben C, Benjamin W. (1985). Scleral contact lenses: preliminary report on oxygen-permeable
materials. Contact Lens J.

[5] Bleshoy H, Pullum KW. (1988). Corneal response to gas-permeable impression scleral
lenses. J Br Contact Lens Assoc.

[6] Giovedi R, Giovedi M. (2003). Coral-Ghanem C, Kara José N, editors.

[7] Barnett M, Carrasquillo KG, Schornack MM. (2020). Clinical outcomes of scleral lens fitting with a data-driven,
quadrant-specific design: multicenter review. Optom Vis Sci.

[8] Barnett M, Fadel D. (2017). Scleral lenses: benefits of toric landing zones. Contact Lens Spectrum.

[9] Consejo A, Behaegel J, Van Hoey M, Iskander DR, Rozema JJ. (2018). Scleral asymmetry as a potential predictor for scleral lens
compression. Ophthalmic Physiol Opt.

[10] Russell B. (2016). visual rehabilitation with contact lenses for irregular corneal
astigmatism. Can J Optom.

[11] Lipener C, Rosa J. (2023). Clinical experience with adjustable scleral
lenses. Arq Bras Oftalmol.

[12] Barnett M. (2017). The right fit for the irregular cornea: smooth things over with
scleral lenses: new scleral designs can help patients with irregular corneas
stay happy and healthy in contact lenses. Rev Optom.

[13] Esen F, Toker E. (2017). Influence of Apical Clearance on Mini-Scleral Lens Settling,
Clinical Performance, and Corneal Thickness Changes. Eye Contact Lens.

[14] Ritzmann M, Morrison S, Caroline P, Kinoshita B, Lampa M, Kojima R. (2016). Scleral shape and asymmetry as measured by OCT in 78 normal
eyes. Poster presented at the Global Specialty Lens Symposium. 2016.

[15] DeNaeyer G, Sanders DR. (2018). sMap3D corneo-scleral topographer repeatability in scleral lens
patients. Eye Contact Lens.

